# Supplementary material for: E2 Partner Tunes the Ubiquitylation Specificity of Arkadia E3 Ubiquitin Ligase
Source: Cancers (Basel). 2023 Feb 7;15(4):1040. doi: 10.3390/cancers15041040 (PMC9954413; doi:10.3390/cancers15041040)
Supplement: Supplementary file 1 [file cancers-15-01040-s001.zip › cancers-2148865-supplementary File S1.pdf]

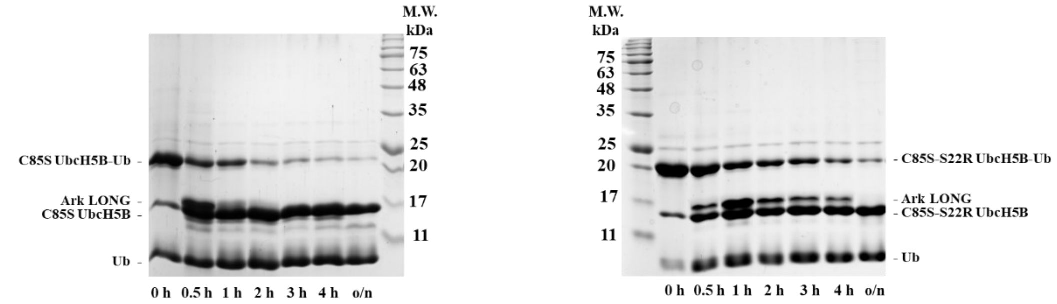

Figure S9. Oxyester hydrolysis assays showing the disappearance of C85S UbcH5B-Ub (left) and C85S S22R UbcH5B-Ub (right) in the presence of AKD LONG, over time.

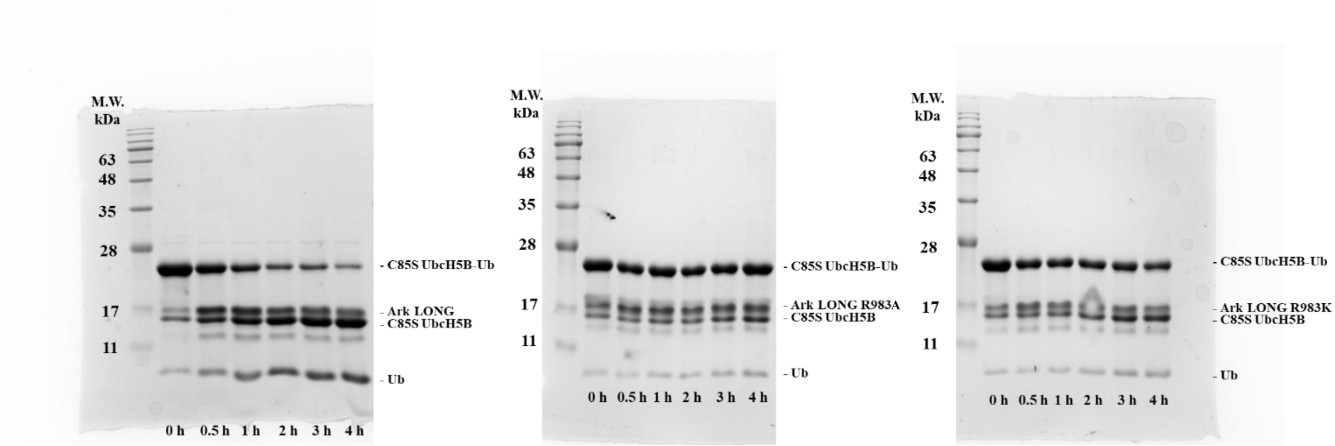

Figure S10. Oxyester hydrolysis assays showing the disappearance of C85S UbcH5B-Ub in the presence of Ark LONG, Ark LONG R983A and Ark LONG R983K respectively, over time.

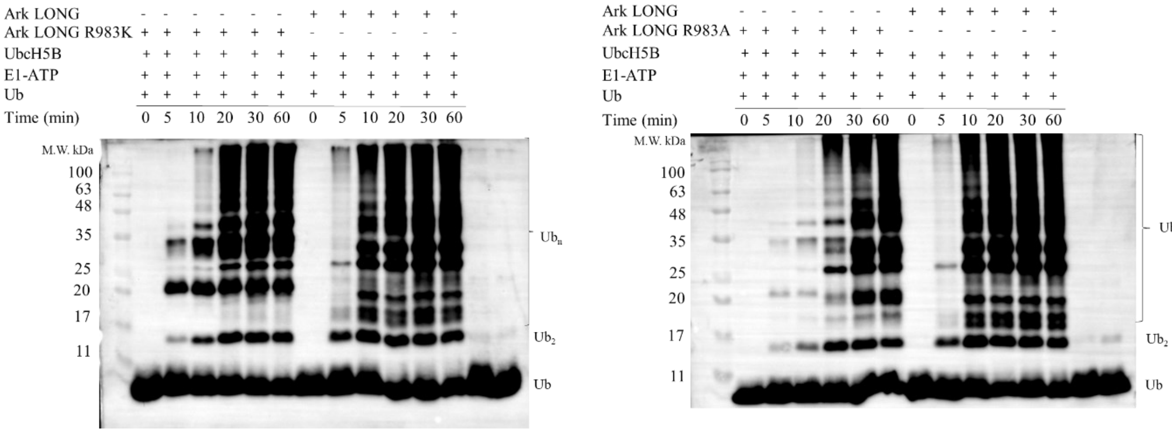

Figure S11. In vitro ubiquitination assays of Ark LONG R983K (left) and Ark LONG R983A (right) in comparison to wt Ark LONG.
