# Supplementary material for: E2 Partner Tunes the Ubiquitylation Specificity of Arkadia E3 Ubiquitin Ligase
Source: Cancers (Basel). 2023 Feb 7;15(4):1040. doi: 10.3390/cancers15041040 (PMC9954413; doi:10.3390/cancers15041040)
Supplement: Supplementary file 1 [file cancers-15-01040-s001.zip › cancers-2148865-supplementary.pdf]

Ark RING 68aa

927 N-KQDGEEGTEEDTEEKCTICLSILEEGEDVRRLPCMHLFHQVCVDQWLITNKKCPICRVDIEAQLPSES-C 994

Ark LONG 119aa

876 N-FRGNFEELIHLEERLGNVNRGASQGTIERCTYPHKYKKVTTDWFSQRKLHCKQDGEEGTEEDTEEKCTICLSILEEGEDVRRLPCMHLFHQVCVDQWLITNKKCPICRVDIEAQLPSES-C 994

Figure S1. Ark RING and Ark LONG C-terminal constructs of Arkadia E3 ligase. Zinc binding amino acids are colored red.

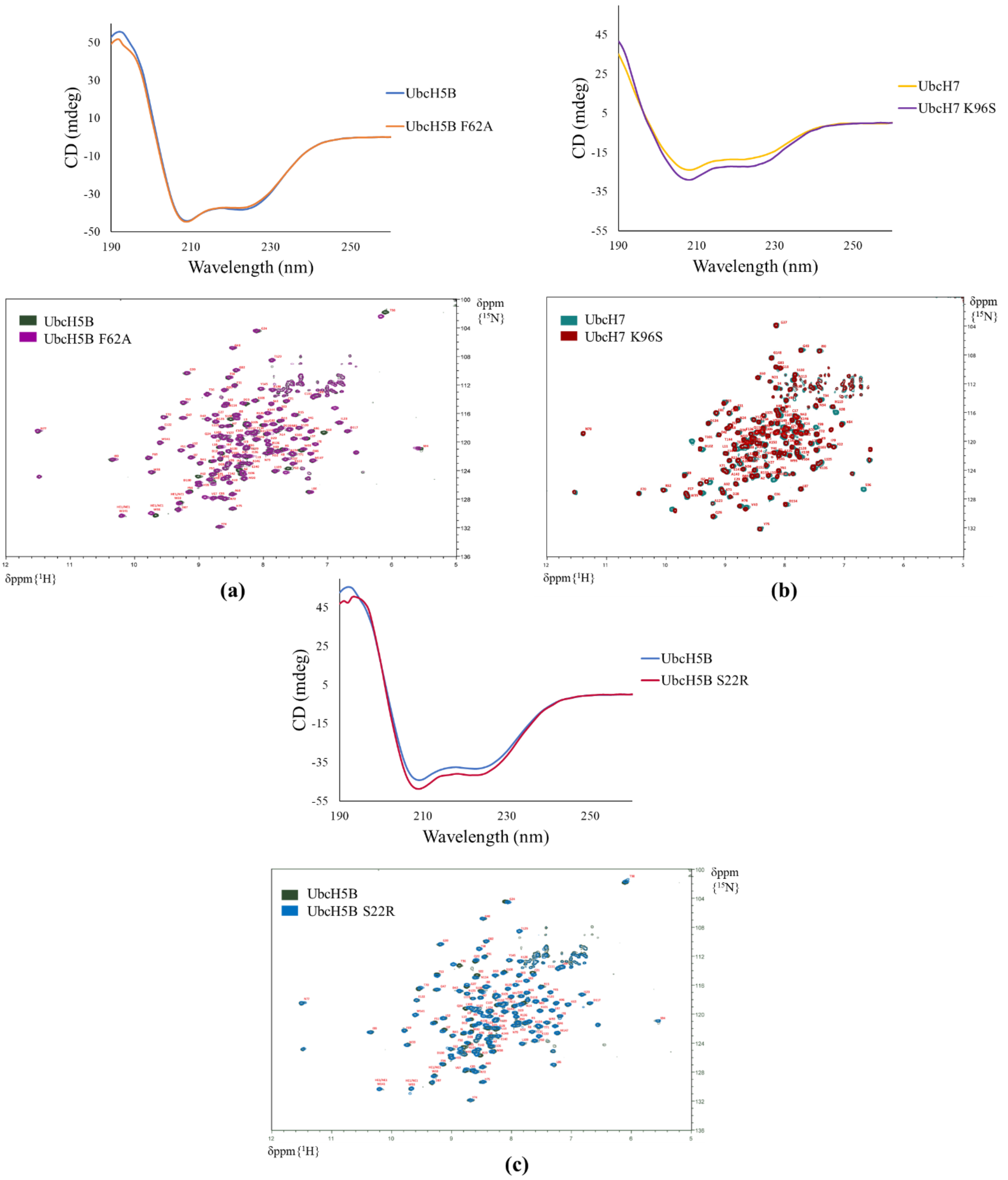

Figure S2. **(a)** (top) CD spectra of wt UbcH5B and UbcH5B F62A. (bottom) Overlay ^{1}H-^{15}N HSQC spectra of wt UbcH5B and UbcH5B F62A. **(b)** (top) CD spectra of wt UbcH7 and UbcH7 K96S. (bottom) Overlay ^{1}H–^{15}N HSQC spectra of wt UbcH7 and UbcH7 K96S. **(c)** (top) CD spectra of wt UbcH5B and UbcH5B S22R. (bottom) Overlay ^{1}H–^{15}N HSQC spectra of wt UbcH5B and UbcH5B S22R.

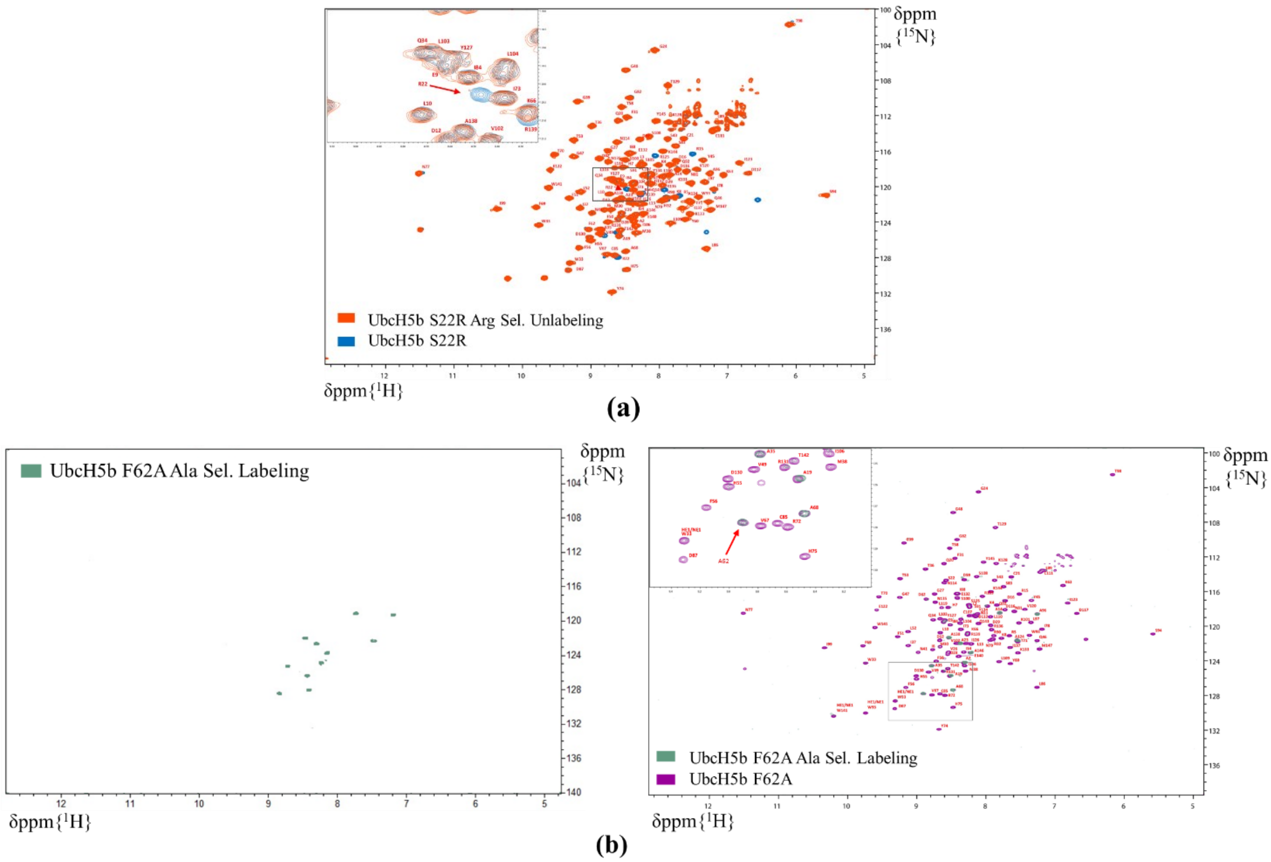

Figure S3. **(a)** Reverse labeling of 15N UbcH5B S22R. **(b)** Selective 15N labeling of UbcH5B F62A with 15N-Ala.

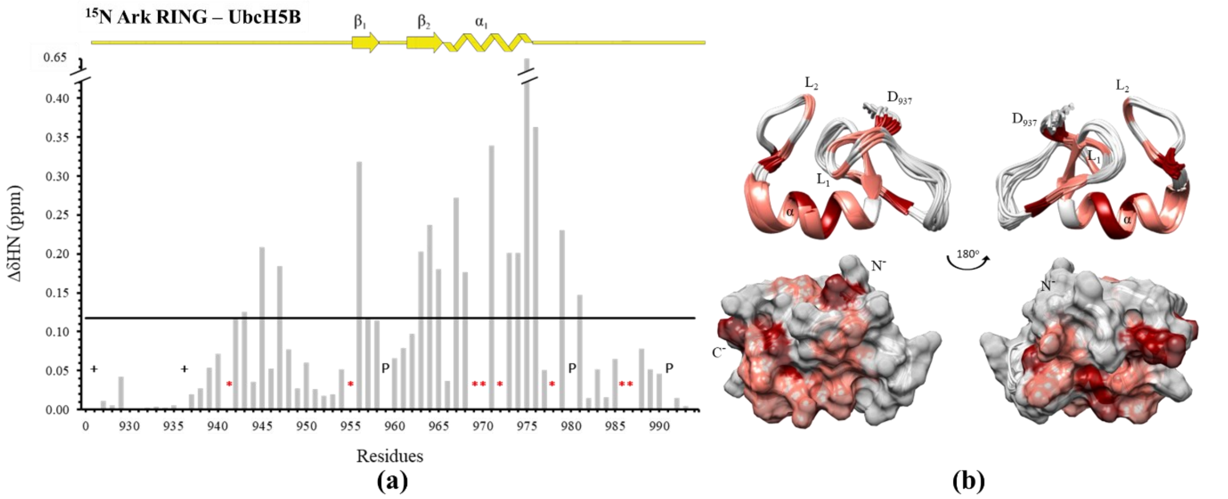

Figure S4. (a) Diagram of the total CSPs measured at 1:2 molar ratio (saturation point) for 15N Ark RING/14N UbcH5B,*: represents disappeared residues, +: represents residues with no information. **(b)** Ark RING mapping (PDBid: 2KIZ) after addition of UbcH5B (residues that disappeared during the interaction are colored red, while residues that exhibited fast exchange interaction are colored coral).

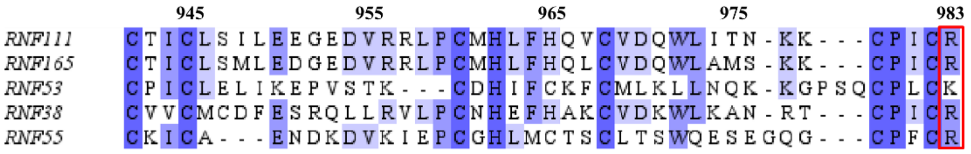

Figure S5. Sequence alignment of the RING domains of different E3 ubiquitin ligases numbered according to Arkadia/*RNF111*. The conserved arginine is highlighted with red box.

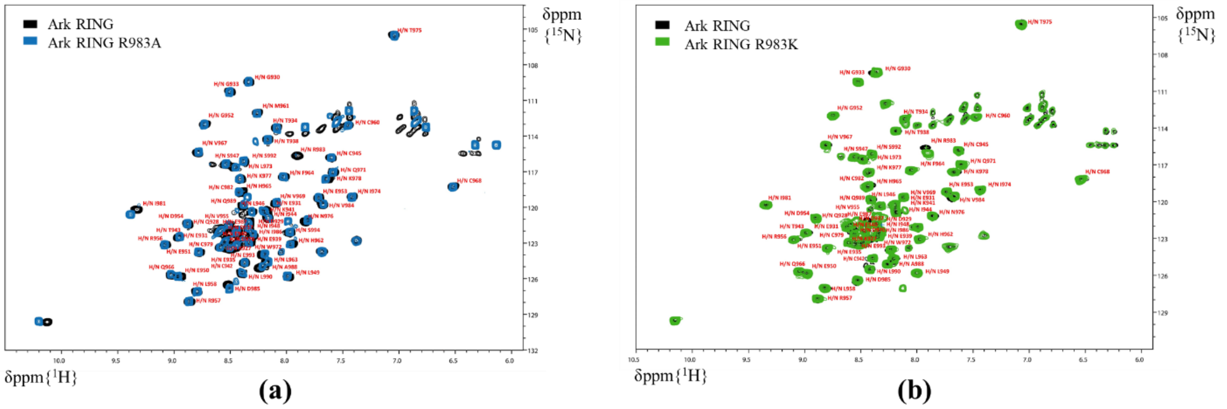

Figure S6. **(a)** Overlay ^{1}H–^{15}N HSQC spectra of Ark RING R983A and Ark RING. **(b)** Overlay ^{1}H–^{15}N HSQC spectra of Ark RING R983K and Ark RING.

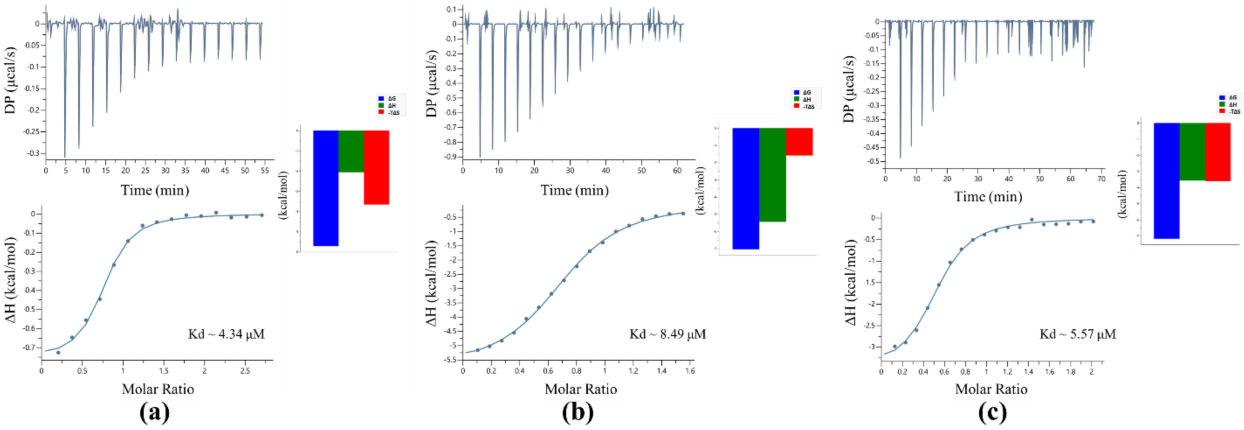

Figure S7. **(a)** ITC data for the titration of wt UbcH7 into Ark LONG. **(b)** ITC data for the titration of UbcH5B into Ark LONG R983A. **(c)** ITC data for the titration of UbcH5B into Ark LONG R983K.

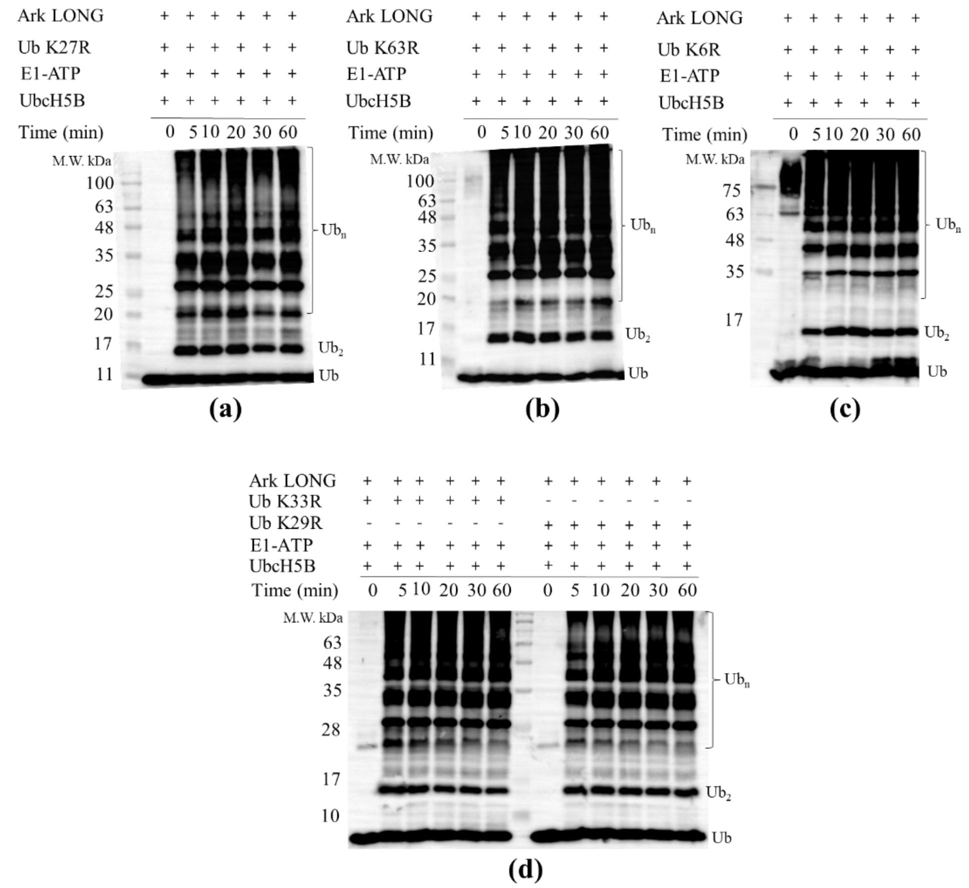

Figure S8. (a) In vitro ubiquitination assays using UbcH5B in the presence of Ub K27R, **(b)** Ub K63R, **(c)** Ub K6R, **(d)** Ub K33R and Ub K29R.
